# Your health is in your mouth: A comprehensive view to promote general wellness

**DOI:** 10.3389/froh.2022.971223

**Published:** 2022-09-14

**Authors:** Antonia Barranca-Enríquez, Tania Romo-González

**Affiliations:** ^1^ Centro de Estudios y Servicios en Salud, Universidad Veracruzana, Veracruz, Mexico; ^2^ Área de Biología y Salud Integral, Instituto de Investigaciones Biológicas, Universidad Veracruzana, Xalapa, Mexico

**Keywords:** oral health, periodontitis, dental caries, wellbeing, comprehensive health promotion

## Abstract

**Background:**

Even though various studies recognize the importance of the oral cavity to have general health, in multidisciplinary professional practice it is almost always excluded and on an individual basis, very commonly neglected. Oral diseases are preventable, still, they are highly prevalent. Although some studies consider oral health within integral health, currently, there is no model in which the mouth is integrated within other levels for the achievement of well-being. The objective of this article was to review the importance of oral health and its connection with well-being and, based on these findings, propose a complex and comprehensive perspective for approach and care.

**Methods:**

The databases MEDLINE, PubMed, and Google Scholar were revised for randomized controlled trials and reviews that included search terms related to oral health and its relationship with the general health in its different levels (physical, psychological, social and environmental).

**Results:**

The review shows that oral health is critical, as the teeth and mouth are not only an integral part of the body, but also, they also support and enable essential human functions. That is, oral health has a multidimensional nature, as it includes the physical, psychological, social, and environmental domains that are essential for overall health and well-being. Likewise, the mouth is the psychological seat of the first physiological needs and emotional gratifications, with it we take a taste of the world around us. Thus, the mouth plays an important role in the feeling of unity and in the constitution of the self. Based on these results we propose an integrative model in which the mouth is the first step for well-being and from this integrative model we build a multidisciplinary approach which could be used in the clinical practice for the promotion of oral care and general health.

**Conclusion:**

The effort on the part of oral health professionals is essential for people's well-being and must be integrated as part of health promotion. Dental treatments alone cannot solve this problem, it requires a comprehensive and approach in which the bio-psychological, behavioral, and socio-environmental determinants are included to face this global oral health challenge. That is, without a comprehensive and multidisciplinary approach to medical science that includes dental and oral health, our public policies cannot provide the best answers to health promotion, disease prevention, early detection, and treatment.

## Introduction

Oral health has traditionally been defined as a disease-free oral state contributing to the normal function of the mouth. Only recently the FDI Dental World Federation (FDI) has recognized that: “oral health is multifaceted and involves the ability to smell, touch, taste, chew, swallow, smile, speak, and conveys a variety of emotions through facial expressions with confidence and without discomfort, pain, or disease in the craniofacial region” (1). Likewise, numerous studies show that the oral cavity plays an important role as it is essential for health, since there is a close relationship between oral diseases and other systemic diseases such as digestive disorders, strokes, diabetes, cardiovascular disorders, metabolic syndromes, adverse events in pregnancy, obesity, Alzheimer's, rare diseases such as Von Willebrand's disease, Chediak-Higashi syndrome, among others (2, 3) and more recently with COVID-19 (4, 5).

Although the pathophysiological mechanism has not been fully explained, oral problems could have serious effects on the body through the dissemination of pathogenic bacteria in blood and bones and by causing a pro-inflammatory state in which systemic diseases could develop (6–17). In fact, since the mouth can be both a window and a gateway to the body, systemic conditions and side effects of medical therapies can lead to early manifestations in the mouth. Unfortunately, the lack of connection between the mouth and the body makes it difficult to recognize these signs and symptoms. On the other hand, there are oral diseases that are not diagnosed in time due to the lack of collaborative approaches, and where the delay can be disastrous, as is the case of oral cancer (18).

Even though the importance of the mouth is recognized, in practice it is not included as part of general health, and oral health care is always neglected. In fact, despite being largely preventable, oral diseases are highly prevalent throughout life and have substantial negative effects on individuals, communities, and society, and are considered a global public health problem; not only because of its increasing prevalence in many low- and middle-income strata of countries (more than 3 billion people suffer from chronic and progressive oral diseases, beginning in early childhood and progressing through adolescence and adulthood), but they are generally connected to social, economic, and commercial changes (18, 19). These diseases are closely related to socioeconomic status and social determinants. For example, increased consumption of free sugars is causing an increase in dental caries, as well as other non-communicable diseases such as obesity and diabetes (18).

Although there are some definitions that place oral health within general health (1), there are, as well some approaches that show its relationship with other diseases and the social determinants of health (2, 3, 18), the socio-biological interactions of the oral diseases (20), and a psychosocial dentistry (21). Nowadays, there is no model in which the mouth is integrated within general health, much less one in which it takes the importance it deserves.

This is due to the lack of a comprehensive and collaborative approach between disciplines, sub-disciplines and professions in both research and clinical practice. That is, unfortunately, the reductionist biomedical/disease-focused model remains dominant, and physicians and dentists (including dental teachers and dental students) still misunderstand the person-centered approach and social dentistry (18, 21). This is very evident in the separation of oral considerations from the rest of general health and from basic science to health care delivery, which has led to a lack of translation from basic science to clinical care and to policies that can reach communities to achieve real advances in health, and consequently oral health and general health systems are ineffective, fragmented, and costly (18).

This separation has also led to the general perception that oral health is somehow less important to general health and well-being and is therefore not given priority in professional training in the health sciences and in dentistry, public policy, or health care delivery. This has resulted in significant disparities in oral health and access to care beyond those seen in other areas of health, disparities that could, in part, be ameliorated by integrated approaches to science, policy, and health care (18).

Given the importance of oral health for the body and the high prevalence of oral diseases, it is necessary to provide evidence that supports the need to integrate oral health care within health promotion, and thus favor wellness.

In this review, we begin by presenting the global public health problem generated by oral diseases and the need to fight them, as well as the findings that show their importance for general health and their levels of interaction, to later propose an integrative model of the mouth with all the elements considered in isolation in a single conceptual approach. Finally, based on this integrative model, a mouth care protocol is proposed in which, the achievement of oral health, well-being, and the early diagnosis of oral and systemic diseases in the dental office can be promoted.

Therefore, the aim of this review article is to document the importance of oral health and its connection with well-being and based on these findings, propose a complex and comprehensive perspective for approach and care.

## Methods

### Search strategy

The narrative review presented below is based on material derived from a literature search conducted using three computerized databases (MedLine, PubMed, and Google Scholar). We attempted to identify all the literature that documented the associations between oral health and general health and that were published in English and Spanish in peer-reviewed journals between January 1995 and December 2020. We searched for the following combination of keywords that covered the outcome of the interest (Table 1) on the following topics:

1. Oral Health
2. Oral Disease
3. Health, Wellbeing, General Health
4. Health promotion and education on Oral Health

**Table 1:** Keywords, inclusion, and exclusion criteria.

Association level	Oral health vs. physical health, oral health vs. psychological, oral health vs. socio-environmental.
Keywords	Oral epidemiology, oral health, oral disease, dental caries, plaque, periodontitis, oral cancer, tooth loss, oral disease progression, malocclusions, stomatognathic system, body and systemic diseases, immunity, Ph, microbiome, epigenetic, psychological personality, behavior, stress, gender, age, socioeconomic status, income, education, environment, integral health, quality of life, health promotion, health determinants.
Study design	No restrictions on study-designs were applied
Inclusion criteria	Empirical studies, English and Spanish language, human studies, including at least one level of association.
Exclusion criteria	Non-empirical studies. Studies that did not include the relationship of the oral health and integral health.

### Review strategy

Abstracts and articles were read because of their relevance to the research question. Articles were selected for review if they met the topic-related criteria of the association of the oral health and general health.

Study characteristics, methodological details and results of statistical analyses were extracted from our 63 articles and entered into a spreadsheet to facilitate data sorting and review. 46 articles that met our inclusion criteria (Table 1; empirical studies, English and Spanish language, human studies, including at least one level of association) were relevant to our research questions of whether there were associations of oral health in human health and development.

## Epidemiological overview of oral health

As the Global Burden of Disease (GBD) study has shown repeatedly, oral health represents the most neglected challenge in the population's health worldwide (19). To that respect, it has been estimated that around 3 billion people around the world lived with dental conditions, among which were predominantly, untreated dental cavities in primary and permanent dentitions, severe periodontal disease, edentulism (complete loss of teeth) and severe tooth loss (with between 1 and 9 teeth remaining) and by 2017 there was only a small decrease of these dental conditions in developed countries (18, 19).

On the other hand, a worldwide longitudinal study that analyzed oral health between 1990 and 2010 found that untreated caries in primary teeth was the tenth most prevalent health condition (affecting 9% of the world's child population); remaining unchanged between 1990 and 2010 (20), as did the age-standardized global incidence (15,205 cases per 100,000 person-years in 2010) (21); and by 2015, untreated caries in deciduous teeth decay among children aged from 1 to 4 (21). Likewise, untreated cavities in permanent teeth were the most common health condition in 2010, affecting 35% of the world's population (2.4 billion people worldwide) (21), with the largest peak being 25 years and a second smaller peak around 70 years. Notably, for 2015, the peak prevalence of untreated dental caries in the permanent dentition was observed at a younger age (in the 15–19 age group) (22).

Regarding periodontitis, this was the sixth most prevalent oral condition in 2010, affecting 10.8% of people (743 million worldwide), and since 1990, the prevalence and incidence standardized by age worldwide have remained stable. In addition, 158 million people (2.3% of the world population) were completely toothless (23).

According to the International Agency for Research on Cancer, lip and oral cavity cancers for 2018 were among the 15 most common types of cancers worldwide, with 500,550 cases and the total number of deaths from lip and oral cavity cancer was 177,384 (67% of deaths in men) (18, 24).

All the above being considered, dental diseases generate an economic burden on society, since they have a direct impact on treatment costs, an indirect impact on productivity losses; and intangible impact on pain, problems with eating, speaking and the expression of emotions such as smiling, actions that can limit social and family activities. For example, in 2015, dental diseases represented $ 356.80 billion in direct costs and $ 187.61 billion in indirect costs worldwide (25, 26). Additionally, oral disease can also intensify the burden of other ailments and thus contribute to the economic health crisis. Likewise, in terms of intangible costs, dental problems can have a negative effect on school performance, possibly worsening social inequalities. Several studies show that cavities and associated oral problems decrease quality of life of the child and their caregivers and also in adults who have little access to dental care. This implies that they must cope with acute and chronic pain, which also reduces their quality of life (18).

Thus, all these epidemiological statistics show that there are clear socioeconomic inequalities that persist with the prevalence of oral diseases, that is, there is a social gradient in oral health. These inequalities have been widely described in literature and some studies in recent years have revealed causal associations between socioeconomic status and dental health (27–30). Particularly, there are extreme oral health inequalities for the most marginalized and socially excluded groups in society, such as the homeless, prisoners, people with disabilities, refugees, and indigenous groups (18).

In this regard, although social determinants of health have been known for some time, the implementation of policies to address these determinants has been slow. Indeed, dental policy makers tend to rely on simplistic interventions as the dominance of the biomedical approach for prevention. This prevails and shapes policies that favor preventive clinical interventions and oral health advice in the office rather than strategies for the entire population.

In this sense, prevention of new cases of dental disease is essential to reduce the burden on health services, and the promotion of oral health aimed at unhealthy behaviors, both have the potential to reduce dental disease and mortality rates attributed to cardiovascular disease, cancer, and diabetes, which are indicators for the evaluation of the Sustainable Development Goals (19).

## The mouth and its importance for well-being

The unfortunate circumstance of not viewing the human being as an integral and integrated whole, represents many difficulties. For example, it has led the human sciences to become fragmented in an intense and profound way (28), leading that the so-called “human behavioral sciences” (psychology, social work, anthropology, etc.) and the “human health sciences” have become increasingly separated, with the aggravating factor that their work has become sectorized to the maximum (28).

An example of this integrality is the stomatognathic system, which is the integrated and coordinated morpho-functional unit that is constituted by the set of skeletal, muscular, angiological, nervous, glandular, and dental structures, organized around the head joints. All these structures are organically and functionally linked with the digestive, respiratory, phonological, and aesthetic-facial expression systems and with the senses of taste, touch, balance, and orientation. The union of all these systems allows to develop the functions of suction and oral digestion, swallowing and verbal communication, affective expressivity (which includes smiling, laughing, orofacial gestures, kissing, among other aesthetic-affective manifestations), alternate breathing and vital defense autonomous actions, like coughing, expectoration, sneezing, yawning, sighing, exhaling and vomiting, essential for the survival of the individual. In this sense, any injury to one of these muscles or the bones where they are inserted, will produce alterations in the position of the head on the vertebral axial axis and, therefore, alterations in the stomatognathic system, in the sense of balance and in the sense of orientation (28).

Therefore, oral health is critical as the teeth and mouth are not only an integral part of the body, but also, they support and enable essential human functions (18). That is, oral health has a multidimensional nature, as it includes the physical, psychological, emotional, and social domains that are essential for overall health and well-being. Oral health is subjective and dynamic, allowing the individual to eat, speak, smile, and socialize, without discomfort, pain, or embarrassment. For all this, good oral health reflects the ability of an individual to adapt to physiological changes throughout life and maintain his/her teeth and mouth through independent self-care (18). In addition, oral pathologies, may be the key to some systemic diseases (1, 2, 18), as a wide range of diseases and disorders affect the soft and hard tissues of the mouth, including several craniofacial disorders, congenital anomalies, injuries, and various infections.

Likewise, as proposed by Rojas-Alcayaga and Misrachi-Launert (29) the mouth is the psychological seat of the first physiological needs and emotional gratifications, with it we take a taste of the world around us. In addition, the mouth provides the first sensations of security, pleasure, satisfaction, and success, bringing all this oral activity to the first perceptions of the self. Thus, the mouth plays an important role in the feeling of unity and in the constitution of the self, since oral functioning serves as a body model in which the psychic experiences that accompany the identification process are based. That is, in the mouth the “self” is represented in an important way, since it clearly marks a boundary between the inside and the outside (as does the skin) and constitutes an element of exchange. The mouth, like it happens with the immune system, makes the “self” and the “other” recognizable, that is, in the mouth the individual is fully represented (29).

The next part of this article provides a brief overview of the importance of oral health in the different levels that integrate an individual’s health: physical, mental and socio environmental levels.

### Oral health and physical health

There is an increasing interest in medical research in the field of dentistry, since various systemic diseases (including rheumatoid arthritis, adverse pregnancy events, cardiovascular disease, diabetes, colon and pancreatic cancer, digestive diseases, obesity, liver diseases, neurodegenerative diseases such as Alzheimer's, among others) (1, 2, 17, 30) and some infectious diseases such as COVID-19 (4, 5), have been associated with the presence of oral diseases. For example, numerous studies tend to analyze all complications from periodontal disease, since, by sharing a common inflammatory pathway, periodontal disease is associated with other chronic diseases, such as diabetes, cardiovascular disease, and dementia (18). In addition, controlling diabetes is difficult for patients with periodontal disease, and therefore patients suffering from periodontitis and diabetes simultaneously are at risk of developing cardiovascular, renal, and retinopathic diseases (2).

On the other hand, the presence of some bacteria responsible for periodontal disease has recently been reported in the brain tissue of patients suffering from Alzheimer's disease (17).

In this regard, within the fields of interest that relate oral disease to systemic diseases is the oral microbiota, which is one of the most complex in the human body. The human oral cavity comprises several different habitats, including the teeth, gingival sulci, tongue, hard and soft palate, and tonsils, which form a species-rich, heterogeneous ecological system, and act as the tube connecting the exterior and the digestive tract and respiratory tract of the human body, which provides the proper conditions for the colonization of microorganisms (31).

The interaction of various oral microorganisms helps the human body against the invasion of undesirable stimuli abroad. This is because, in a similar way to the intestinal microbiome, it helps train the immune system, keeps dangerous colonizers away and produces small molecules that nourish the cells that line the mouth. However, the microbiome can become an enemy if the host-microbe relationship becomes unbalanced. Therefore, a thorough understanding of the factors that shape the human microbiome is key to prevent and control diseases (32).

The microbiology of a healthy individual has been associated with many factors, such as: (1) Time, since our microbiota is not only personal, but it varies systematically in the same person according to body habitats and time, in fact, it has been seen that the microorganism communities in the oral cavity are the most unstable. (2) Age, bacteria vary not only in time but also according to different age groups. (3) Diet, it has been seen that the oral microbiotic ecosystems of people on fashion are less diverse, which could contribute to chronic oral disease in post-industrial lifestyles. In particular, large differences have been found between hunter-gatherers, traditional farmers, the Western diet, and vegetarians in terms of the oral microbiota. For example, some oral microorganisms have been found in hunter-gatherers, showing that eating too much meat predisposes a high risk of oral disease; while for vegetarians, the composition of the oral microbiota is significantly altered; (4) Other factors with breastfeeding as a baby, gender and education level also generate variations in the microbiome (30, 31).

On the other hand, in recent years, with the completion of the Human Microbiota Program, people have become more and more aware of oral microbes, but little has been done to analyze oral microbiota, even though it is known that it can produce metabolites in the mouth that can lead to the development of a variety of oral diseases such as tooth decay, periodontal disease, and oral cancer (31).

Nevertheless, there are numerous microorganisms in the mouth, including bacteria, viruses, and fungi, bacteria are the main inhabitants of the oral cavity. This microbe can disrupt the interaction between the microbiota and the host mucosa by modulating the innate immune system, promoting inflammation and chronic infections (33).

Some of the described pathways in which the microbiota can generate systemic diseases are the following: (1) The oral microbiota enters the intestinal tract causing a micro-ecology imbalance of the intestine which affects the digestive system. (2) Oral microorganisms, especially periodontitis pathogenic bacteria, can enter the systemic circulation through the periodontal blood, thus acting throughout the body. (3) The metabolites of the oral microbiota enter the bloodstream and systemic circulation, which generates a low-grade inflammatory state. All this promotes the development of various chronic diseases.

Thus, oral microbiomes play an important role in the human microbial community and human health status. Therefore, studies on oral microbiomes and their interactions with whole-body microbiomes in various body sites and varying health conditions are important to our knowledge of the human body and how they affect the improvement of human health.

On the other hand, it is important to consider that oral diseases are chronic and progressive in nature, that is, they have a natural history of the disease, which can develop from pregnancy, lactation, childhood and continue throughout life from adolescence to adulthood (18, 34). For example, there are reports that dental caries could be transmitted vertically from mother to child, when teeth begin to erupt, the oral cavity becomes receptive to the colonization of various bacteria (34). Likewise, there is some evidence that children whose mothers (or other caregivers) received advice on healthy diet and feeding practice for infants, were less likely to have tooth decay up to the age of six in comparison to those whose caregivers received the usual care (35). Hence the importance of health promotion and of treating mothers before delivery or during the time from delivery until the child has the first tooth at approximately 6 months of age (34).

Moreover, it is well known that the greatest susceptibility to cavities in permanent dentition occurs precisely in the period after tooth eruption, especially in the first year after teeth have erupted. Several studies describe the most important aspects of the complex physicochemical mechanism of de-remineralization of dental enamel and are known as the main factors: the inhibitory influence of salivary proteins and fluoride, the anatomical variations of the dental elements, the chemical behavior of phosphates, the importance of charge and diffusion coefficients on the gradient. The stability-instability of the system depends on the pH of the medium (it has been shown that decalcification of the tooth is accentuated when the pH falls below 5.5), on the concentration of fluorides (teeth with fluoride enamel are much more resistant to descaling) and ionic strength. Both *in vitro* and *in vivo*, the persistence of acidity favors dissolution, while the reduction of the exposure time stimulates remineralization. In addition, many authors agree that it is fundamental for the resistance of enamel to acid dissolution the period in which the dental structures are in formation (where the mother's nutrition plays a fundamental role) and later the period of calcification in which breastfeeding is very important. This shows that calcium concentrations are significantly higher in children who are breastfed, since their mothers ingest more amounts of energy, total protein and carbohydrates compared to mothers who do not breastfeed. Furthermore, children classified as malnourished present structural alterations in dental tissues with a marked dependence on tooth eruption and the presence of caries as a result of nutritional status (34). Therefore, although the cultural influence of the population and oral health professionals do not consider the need for dental treatment before the deciduous dentition is completed or until three years of age, when the child presents the psychological conditions that make attention possible; given the new knowledge about the importance of caring for deciduous teeth in the dental arch until their exfoliation, it is necessary for health professionals, in the public or private health network, to recognize that the promotion and prevention of oral health in infants, in the earliest stage of life, preferably from the prenatal stage (35).

But not only oral diseases come from earlier stages of life, but also from an inherited susceptibility. On the subject of this, there are several reports in which oral diseases can be transmitted epigenetically to the following generations, proposing that dental anomalies are the result of genetic-epigenetic interactions (36–38). This is because environmental and lifestyle (diet, medications, physical activity, mental or physical stress, traumatic events, environmental toxins, and addictive substances such as nicotine and alcohol), can interact with the genome and influence through epigenetic changes in developmental abnormalities, mental or behavioral disorders, infectious or inflammatory changes, precancerous or cancerous conditions, and many other abnormalities (37). For example, various nutritional factors such as folate, vitamin B12 and vitamin A can cause epigenetic changes. Furthermore, smoking also causes hypomethylation and hypermethylation changes in DNA. Therefore, some exogenous factors such as diet, smoking, environment, bacteria, inflammation, and age can affect oral health by causing epigenetic changes (36). In addition, epigenetic mechanisms play an important role in gene expression during dental development (from the sixth week of gestation to approximately the twentieth year of life), therefore, during this long period of time, the processes of the normal development can be significantly disturbed and influence the progress of various oral diseases. Therefore, an understanding of epigenetic mechanisms is essential and has strong implications for research and practice in the dentistry area (37).

### Oral health and psychological health

Several studies have shown how different personality characteristics of individuals can affect oral health (39–44); since personality disorders may increase the risk of oral disease and alters the attitude of individuals towards the disease (41). That is, some personality characteristics such as anxiety, stress, depression in daily life, self-esteem, behavioral patterns, and lifestyles, have been related to oral health behavior. In fact, brief cognitive interventions can alter attitudes and values of personality traits, which could be useful in preventive dentistry (41).

In this regard, research has indicated that at least three processes are implicated. Firstly, personality traits influence oral health. Secondly, certain personality traits are associated with unhealthy behaviors that can affect oral health. Thirdly, personality characteristics can alter the way individuals respond to (interpret) symptoms and thus create their disease state (40). Thus, for example, various psychological factors have the potential to alter gingival tissues and host immune responses, resulting in more severe periodontal disease expression (41). In addition, reports have shown that mental stress (allostatic load) can generate an increase in total saliva and changes the characteristics of saliva (i.e., protein concentration, cortisol levels, pH, etc.). This is of great importance, since salivary proteins support the ecology of the oral cavity, increase the defensive mechanisms in the mouth, determine the chemical and physical properties of saliva (such as viscosity and lubrication), in addition to having special functions not only for the health of the oral cavity but also for microorganisms, including nutrition, survival and colonization as well as for the adhesion and aggregation of microorganisms; and at the same time fluoride in saliva is essential for the balance between demineralization and remineralization of enamel. Therefore, the changes generated by stress in saliva increase susceptibility to oral diseases, particularly the progression of dental caries (45).

Various studies also propose that there is an impact of the general personality of an individual on the oral hygiene status of the person. In this regard, most of the patients who do not attend the dental clinic have been reported to belong to extroverted and neurotic personality types. In addition, high scores of the Simplified Oral Hygiene Index, the Plaque Index, the Gingival Index have been correlated in people with neuroticism. Also, individuals with a dominant aggressive personality character show bruxism, stressed individuals interpret oral symptoms more adequately due to their own character, and people with low extraversion scores are more likely to smoke, which may predispose them to poor oral health and a consequent periodontitis (41).

But not only personality characteristics influence oral health. Oral disease also has serious repercussions on mental health. For example, malocclusion, a common problem of tooth misalignment, not only influences the patient's body image and oral function, but also leads to serious mental and psychological disorders, thus, malocclusion reduces the quality of life, which also increases with the severity of misalignment (42). Individuals with better dental appearance have generally higher self-esteem and social acceptance than those with dental problems, which indicates that an unattractive dental appearance has negative social impacts on people, in addition, people with poorer self-reported oral health are more prone to anxiety, stress, and depression (42). These findings indicate that the severity of malocclusion is associated with quality of life related to oral health, although, even with the same degree of malocclusion, the impact of self-perceived oral conditions on well-being differs according to the individual's psychological state (42). That is, under the same conditions, people with personality traits of aggressiveness, coldness, anxiety, and stress are more influenced psychologically and socially by malocclusion.

On the other hand, parafunctional habits are frequently observed in the general population and can cause damage to the teeth, the masticatory system and/or the joints when they exceed the physiological tolerance of the individual and the structural tolerance of the masticatory system (43). In this regard, it has been reported that psychological factors are an important component in the etiology and maintenance of parafunctional and temporomandibular habits, showing that the personality characteristics of the patient that reflect their behavior and responses to stress and anxiety are important psychological factors for these disorders. Thus, subjects with higher scores for hysteria, depression, and anxiety are more likely to experience facial pain and have tired jaws (43).

Regarding behavior, personality and oral health, Myers et al. (46), hypothesized that repressors compared to non-repressors would report better self-care behavior for dental hygiene, but worse health-care behavior when a dentist is perceived to be in control. When conducting such a study in a group of adults they found that indeed, the repressors compared to all the non-repressive groups reported that they brushed their teeth more times a day and for a longer time and were less likely to forget to brush their teeth. Nonetheless, the repressors informed fewer visits to the dentist and desired more control in dental surgery. Thus, repressors reported significantly better dental self-care behaviors, that is, behaviors under their control, but were significantly poorer in behaviors that were not under their control (39).

Moreover, although there is little research on the correlation between quality of life, oral health and psychological state, this information is essential for the design of better dental treatments, since personality traits can psychosocially affect self-reported oral health, suggesting that dentists should consider psychosocial status in treatment, in addition to normative clinical measurements (42).

Finally, recently it has been shown that chewing not only enables the mechanical crushing of food and aids digestion but also stimulates the central nervous system (particularly in the hypothalamus and hippocampus) and homeostasis, which increases the temperature in the brain, improves cerebral blood flow, and activates the metabolism in the brain. Thus, tooth loss could also be a risk factor for cognitive decline and various forms of dementia, including Alzheimer's disease, and other causes of, such as Parkinson's disease (47).

### Oral health and socio-environmental health

Oral conditions disproportionately affect impoverished and socially disadvantaged members of a population. There is a strong and constant social gradient between socioeconomic status and the occurrence and gravity of oral diseases. In this way, oral diseases can be considered as a sensitive clinical marker of social disadvantage, being an early indicator of poor health in the population linked to deprivation (18, 48). However, it is not clear why people with low socioeconomic status are more susceptible to poor health. Possible explanations include the adoption of unhealthy eating habits and lifestyles by this segment of the population and the exposure to several factors that undermine health, or a combination of these factors. In addition, lower educational attainment is associated with poor oral hygiene, tooth loss, and periodontal disease, and should be considered when assessing risk and planning appropriate preventive measures (49). Thus, this caries phenomenon, can be influenced by social, genetic, biological, and cultural factors (49).

On the other hand, the results of programs aimed at reducing costs or access barriers show that these obstacles are simply part of this multidimensional problem of dental health. The study of the relationship between geographic location and health has received increasing attention in the medical literature and several studies in the field of health geography have quantified the association between health and disease characteristics and socioeconomic characteristics. These studies indicate that even in a largely neglected population there were significant differences in terms of caries experience associated with specific geographic factors. Socioeconomic status and family wealth are similar throughout the study area, and the region's standard of living is considered poor by Western standards. Settlements in mountainous areas in many parts of Latin America have consistently followed a pattern in which groups at the lowest socioeconomic level are relegated to the least desirable lands, further from rivers and plains. If the range of socioeconomic status is as narrow as we think it is, and if the population is ethnically homogeneous (thus minimizing the role that genetic variability plays in caries vulnerability), then it is possible that the observed differences in the experience of cavities are attributable to increased exposure to caries-inducing agents and/or varied access to dental care. In addition, the variables presence of piped drinking water (due to its fluoride levels) and the proportion of paved roads were associated with oral health (49).

Likewise, oral diseases and oral health inequalities are directly influenced by broader social and commercial determinants, which are the underlying factors of poor oral health in the population (18). Although the social determinants of health have been well known for some time, the implementation of policies to address these determinants has been slow. The dental public health community has been advocating for the importance of integrated bottom-up and community-based approaches; however, oral health care and disease prevention approaches still operate largely in an unintegrated dental silo. Dental policy makers tend to rely on simplistic subsequent interventions; in part, due to the dominance of a clinical interventional philosophy and due to the challenges of generating evidence of efficacy for more complex interventions. Therefore, the biomedical approach to prevention prevails and shapes policies that favor the delivery of preventive clinical interventions and oral health counseling in the office, rather than initial strategies for the entire population (18).

On the other hand, there are reports, worldwide, that show there has been a constant general increase in the production and consumption of sucrose (from sugar beet and sugar cane), the sweetener most available since the 1980s. Consequently, the prevalence of tooth decay is increasing at the same time as marked increases in consumption of sugars including sugary drinks have been reported. This, together with the fact that dental caries are not detected or treated for long periods of time in individuals with poor access to dental care is a possible explanation for the above-average dental caries experience among these groups. In this sense, caries have been described as a “sentinel disease” for other pediatric conditions, with dental care being the most frequently unmet need. That is, this demographic and nutritional transition has been characterized by some adverse changes in diet, but also in physical activity and health (18). Thus, recently, more emphasis has been placed on the combined influence of lifestyle, education, psychosocial factors, and socioeconomic factors rather than the usual risk factors in the treatment of chronic diseases. Age, sex, smoking, anxiety, stress, depression in daily life, self-esteem, self-competence, body image, caring, and protection were positively related to oral health behaviors (49).

## Towards a model for holistic health through the oral cavity

**Figure 1:**
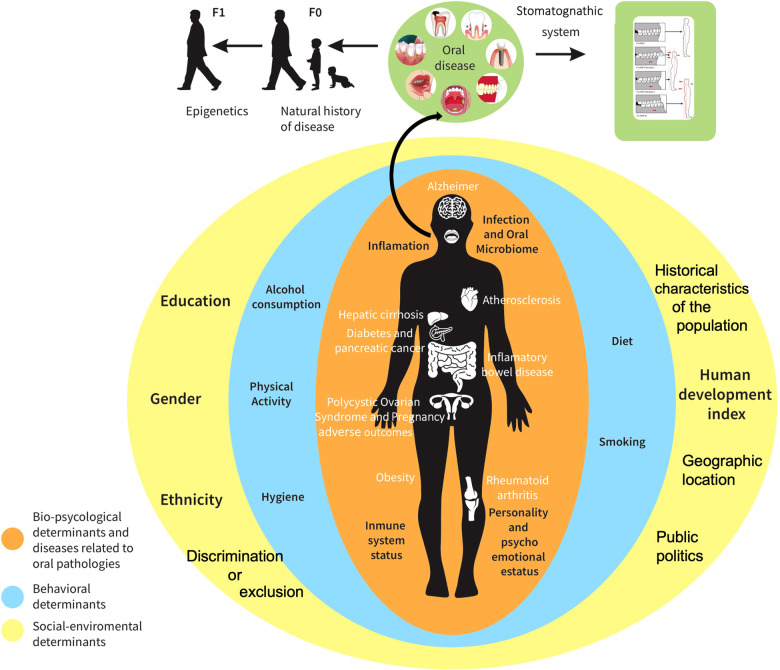
Model for holistic health through the oral cavity. The figure shows the interdependent relationships between general health and oral health integrated in a model of “layers” in which the factors/determinants that compose it interact, directly or indirectly, within and between the different levels of the organization. The first layer in orange are the bio-psychological determinants and diseases related to oral pathologies; the second layer in blue are the behavioral determinants, and finally the third layer in yellow are the socio-environmental determinants. In addition, the model includes in green the relationship between the different oral diseases and its natural history of the disease which are heritable through epigenetics mechanisms, and the relationship of the stomatognathic system with the musculoskeletal system.

Currently there are some models that show the importance of oral health for general health, as well as the relationship that the mouth has with the other systems of the body, the emotions and the mind and the social determinants of health (18, 20, 21). However, from our perspective, the concept of health and oral health must be integrated into a model of “layers” in which the factors/determinants (bio-psychological, behavioral, and socio-environmental), the stomatognathic system, the natural history of disease and the epigenetics mechanisms that compose it interact, directly or indirectly, within and between the different levels of the organization (Figure 1). Thus, in our model, oral health is not only part of general health but a fundamental determinant of general health; since, when there is an oral pathology, there are imbalances throughout the body. For example, various systemic diseases such as Alzheimer's, atherosclerosis, inflammatory bowel disease, rheumatoid arthritis, obesity, polycystic ovarian syndrome and adverse pregnancies, diabetes, pancreatic cancer, or liver cirrhosis have been related to oral pathologies such as caries, periodontitis, oral cancer, among others (2–17, 30, 31). Some of the mechanisms that have been related to this association of oral pathologies and systemic diseases include inflammation, changes in the microbiome of the mouth and its consequent infection, alterations in the immune system and variations in mood and personality (30–32, 39–46). For example, as it was shown previously numerous studies tend to analyze all complications from periodontal disease, since, by sharing a common inflammatory pathway, periodontal disease is associated with other chronic diseases, such as diabetes, cardiovascular disease, and dementia (18). In addition, controlling diabetes is difficult for patients with periodontal disease, and therefore patients suffering from periodontitis and diabetes simultaneously are at risk of developing cardiovascular, renal, and retinopathic diseases (2). On the other hand, the presence of some bacteria responsible for periodontal disease has recently been observed in the brain tissue of patients suffering from Alzheimer's disease (17). Furthermore, the oral microbiome changes with time, age and the diet, and these changes could also have profound impacts on human health. Additionally, several studies have shown how different personality characteristics of individuals can affect oral health (39–44) and vice versa, oral disease also has serious repercussions on mental health (41). That is, some personality characteristics such as anxiety, stress, depression in daily life, self-esteem, behavioral patterns, and lifestyles, have been related to oral health behavior. Besides, reports have shown that mental stress (allostatic load) can generate an increase in total saliva and changes in the characteristics of saliva (i.e., protein concentration, cortisol levels, pH, etc.).

It should be noted that this association goes in both ways, that is, the mouth being a system within another more complex system, changes in the body, mind and emotions also generate alterations in oral health (39–46).

On the other hand, the mouth being part of the stomatognathic system, it has been reported that malocclusions have great impacts on the musculoskeletal system of the body and vice versa (28). For example, there are reports that in a patient who is considered normocclusal, a podiatric defect such as flat feet will produce a forward shift of the center of gravity with permanent head tilt, which will be reflected in an anterior projection of the head, in which the mandible or functional pseudopromandibulism will have a forward and downward displacement of the mandibular condyle. To compensate for this position of the head and jaw and for the bipupillary line to return to horizontal, it is necessary to raise the forehead by contracting the muscles of the back and the sides of the neck (including the trapezius and the sternocleidomastoid), which increases the lordosis of the cervical spine, allows the return of the condyles of the mandible to their functional centric position and the normalization of the inclination of the otic lymph in the semicircular canals, the saccule and the utricle. But, by leading to a permanent contraction of the muscles of the back and the sides of the neck, and a hyperextension of the supra and infrahyoid muscles, cervicogenic pain syndromes can be originated. In this sense, even in patients in whom the stomatognathic system is the only concern of the stomatology professional, the influence of the body scheme has great repercussions, therefore, only through an integral and integrative vision of the human being the solution to the problems of stomatognathic dysfunction may initiate (28). This is of great relevance since depending on the severity of the muscular alterations and their location, alterations of the stomatognathic system, the sense of balance and the sense of orientation can go unnoticed, especially since oral health professionals are not used to looking for and correlating these findings.

But not only the psycho-physiological changes at a given moment are important in oral health and general health. The history of the diseases that an individual has had throughout their life, those of particular importance occurring in childhood and the habits that were acquired in these early stages of life are also important. For example, there is some evidence that children whose mothers (or other caregivers) received advice on a healthy diet and feeding practice for infants were less likely to have tooth decay up to the age of six in comparison to those whose caregivers received the usual care (35). Furthermore, all this history of the diseases has been reported to be heritable up to the next three generations through epigenetic mechanisms (34–38). To that respect, epigenetic mechanisms play an important role in gene expression during dental development (from the sixth week of gestation to approximately the twentieth year of life), therefore, during this long period of time, the processes of the normal development can be significantly disturbed and influence the progress of various oral diseases (37).

Finally, the determinants of oral and general health do not remain with the individual, but also the sphere of the determinants of behavior (diet, smoking, the consumption of alcoholic beverages, hygiene and physical activity) and the sphere of the socio-environmental determinants (ethnicity, gender, education, the human development index,^1^ discrimination or exclusion, geographical location, public politics, historical characteristics of the population) have important bi-directional repercussions on comprehensive health and therefore should be considered in the clinic and in health promotion, in which the mouth has a preponderant role (18, 41, 49).

For example, the burden of oral disease is particularly high among disadvantaged and poor population groups, indicating that social stratification has detrimental effects on both oral and general health (50). That is, oral health disparities adversely systematically affect people who have experienced greater obstacles to health based on the economic position, racial or ethnic group, gender, age, physical disability, etc., linked to discrimination or exclusion (50). In addition, these social determinants of health tend to co-vary with an “eco-social” framework. Thus, it is now understood that certain social environments cause disease through the influences of social and environmental factors on people's health and disease. In fact, recent evidence shows possible neurobiological pathways that link the socio-economic position with health and disease, in which the chronic stress or the allostatic load is the best known (18, 50).

## Comprehensive care and training with an oral perspective

Just as oral health has usually been considered as a decontextualized part of general health, in routine dental care, and the training of dentists, this disconnection of the mouth is also present.

So much so that historically, the medicine-dentistry dichotomy has existed, originating the teaching of medicine for a human being without a mouth and dentistry for a mouth without a human being (28). As expressed by Barreto (28), this conception is expressed in the daily work of doctors and dentists, in which doctors see the mouth as the access route to the pharynx rather than as an integral, very special part of the digestive, respiratory and human oral expression systems; while dentists concentrate their activities on the ideal morphological restoration processes of teeth, while ignoring or minimizing the repercussions that their work has on the other structures of the stomatognathic system and on the rest of the systems that constitute the human being.

That is why it should be kept in mind and constantly emphasized that the human being is an integral and integrated unit in whose operation each and every one of the organs, and systems, has an impact on the functioning of the others, not only on the morphofunctional aspects but also in the psychoneural and psychosomatic, mental and spiritual aspects. This essential integrality of the human being, which makes room for the concept of “holistic”, is continuously manifested in all of his activities and not only in relation to the health/disease process (28). In addition, since the mouth is the psychological seat of the first physiological needs and emotional gratifications, and in the feeling of unity and in the constitution of the self, the difficulty of exposing this area of the body as intimate to another person is understandable, as it is the dentist, who in many cases is a stranger and who is also going to perform interventions that both objectively and subjectively involve some form of aggression. That is, if it is only physical damage, it is the threat to psychological integrity, to self-perception (29).

**Figure 2:**
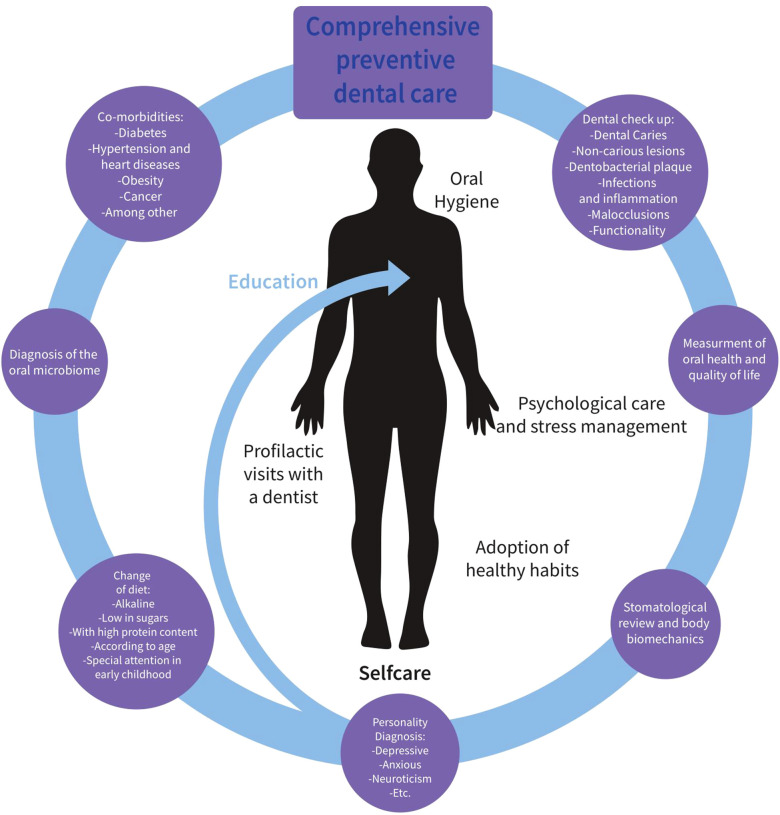
Comprehensive attention model through oral care.

In this sense, it is necessary to train the dentist especially, but also the patient, in comprehensive oral care. Here we propose a model, in which, at least, there are two levels of care (Figure 2); that provided by the dental professional and the part for which the patient is responsible through self-care. In the first level that we have called comprehensive preventive dental care, the dentist, in the first visit, above all, will at least:

1. Do a complete medical history to identify the presence of other systemic diseases (i.e., diabetes, hypertension and heart disease, obesity, cancer, among others)
2. Will measure oral health and quality of life through a validated instrument
3. Review the diet and the presence of unhealthy behaviors (i.e., smoking or drinking excessively) through a guided interview
4. Will identify through validated psychological tests personality characteristics (anxious, depressive, neuroticism, among others) that have been specifically related to oral health
5. Perform a stomatological and biomechanical review of the body
6. Do a general dental check-up (cavities, non-carious lesions, dental plaque, tartar, periodontal disease, infections and inflammation, cancer, malocclusions, functionality, etc.)
7. Will take a sample for the diagnosis of the oral microbiome.

Likewise, the dentist will give essential guidelines to the patient on oral hygiene and healthy lifestyle habits (education). In the second level of self-care, the patient after receiving comprehensive preventive dental care will commit to:

1. Perform oral hygiene according to the parameters taught by the dentist
2. Adopt healthy habits (a balanced and *ad hoc* diet with the foods found proposed by a nutritionist, not smoking, and not drinking excessively, in particular)
3. Seek psychological and stress management help if required
4. Periodically attend prophylactic visits with the dentist.

We believe that this model will allow not only the prevention of many oral and systemic diseases of epidemiological importance, but will also promote health and healthy lifestyles in the population. Thus, it is essential that dental schools, and other health professionals, adopt such holistic and inclusive perspectives.

However, it is important to consider that both the holistic health model and the care model are based on a narrative review of the literature in which there was no rigorous methodology as in the case of systematic reviews. In this sense, there could be certain interpretive biases, so it will be necessary to verify the operability of both models in empirical studies in humans that show the effectiveness of their associations and their use in the health promotion and prevention of the disease.

## Conclusion

Based on the data and the proposals made in this article, it is clear that the oral cavity is not only important for general health (a unique and invaluable treasure) but also a way to promote health. In this regard, in-depth research in medicine, biology, psychology, and oral pathology, in addition to the prevention of some systemic diseases, will lead to the early diagnosis of other diseases in the medium term, including rare diseases.

In addition, health systems and services must integrate general health with oral health, not only because dentists and hygienists can promote oral health and general health at the same time, and dentists therefore need to be better integrated into health systems. Thus, health systems and services must promote general health and oral health, thus favoring the introduction of at least 3 new areas in dentistry: (1) interdisciplinary work and cooperation between various sectors such as education, work, sports and recreation, commerce, agriculture, food, and culture, sharing efforts, strategies, and resources for care, promotion, education, and health care. (2) Formation and participation of dentists in the basic health teams, where they team up with other professionals and contribute their knowledge and effort to promote oral and general health for the population. (3) Change care models from an individual-curative model to an integrative model focused on health promotion, relying on community interventions, primary health care and the adoption of healthy lifestyles to reduce both exposure and risk. We believe that through this approach it will then be possible to respond to the population's growing oral and general health needs, since the current organization of health services in dentistry continue to favor individual care with a capitalist vision and, therefore, a low population coverage.

A reorientation of dental services is therefore required to promote better general and oral health in populations. This necessarily requires modifying the teaching of Dentistry and generating the necessary changes in the professional work and in the relationship of dentists with other health professionals, and above all changes in the relationship of professionals with the community. The workplace for most dental professionals shifts from dental clinics to homes, schools, factories, neighborhoods, and to the rural sector. In addition, dentists would not only improve oral health, but would also include the improvement of general health in their portfolio of services and would team up with other professionals in family-community health and primary health care strategies.

## Footnotes

^1^Characteristics of the Human Development Index (HDI): (1) Health: measured according to life expectancy at birth. (2) Education: measured by the adult literacy rate and the combined gross enrollment rate in primary, secondary, and higher education, as well as the years of compulsory education. (3) Wealth: measured by GDP per capita PPP in international dollars.
